# Non-Motor Symptoms of Parkinson’s Disease and Their Impact on Quality of Life in a Cohort of Moroccan Patients

**DOI:** 10.3389/fneur.2018.00170

**Published:** 2018-04-04

**Authors:** Houyam Tibar, Khalil El Bayad, Ahmed Bouhouche, El Hachmia Ait Ben Haddou, Ali Benomar, Mohamed Yahyaoui, Abdelhamid Benazzouz, Wafa Regragui

**Affiliations:** ^1^Research Team in Neurology and Neurogenetics, Faculty of Medicine and Pharmacy, Genomics Center of Human Pathologies, University Mohammed V, Rabat, Morocco; ^2^Department of Neurology and Neurogenetics, Hôpital des spécialités de Rabat, Rabat, Morocco; ^3^University de Bordeaux, Institut des maladies neurodégénératives, UMR 5293, Bordeaux, France; ^4^CNRS, Institut des maladies neurodégénératives, UMR 5293, Bordeaux, France

**Keywords:** Parkinson’s disease, motor symptoms, non-motor symptoms, quality of life, Moroccan patients

## Abstract

**Background:**

Non-motor symptoms (NMSs) are a real burden in Parkinson’s disease (PD). They may appear in early pre-symptomatic stage as well as throughout the disease course. However, their relationship with the deterioration of the patient’s quality of life (QoL) is still under debate. This study aimed to investigate the prevalence of NMSs and their impact on the QoL in a cohort of Moroccan patients.

**Methods:**

We carried out a cross-transactional study, where a total of 117 patients were submitted to a structured clinical interview and examination investigating motor and NMSs based on common and conventional scales. Motor symptoms were assessed by the UPDRS I–VI during ON condition. The NMSs were evaluated with common scales and their relationship with the QoL was investigated.

**Results:**

The mean patient’s age was 60.77 ± 11.36 years old, and the median disease duration was 6 years [2.5–9.5]. Motor’s phenotype subtypes were the mixed form in 40.2% of patients, akinetic-rigid in 20.5% and a tremor-dominant form in 39.3%. The median Hoehn and Yahr staging was 2 [1–2.5]. Regarding NMSs, the most common were urinary dysfunctions (82.6%), sleep (80.6%), and gastrointestinal (80%) disorders. Other autonomic dysfunctions were also frequent: thermoregulatory dysfunctions 58.6%, cardiovascular troubles 50.9%, and sexual dysfunctions 47.9%. Depression was present in 47.9% and fatigue symptoms in 23.1%. The median score of SCOPA-AUT was 14 [7.75–21.80]. The median PD questionnaire 39-score index (PDQ39-SI) was 23.22% and the most affected dimension was “mobility.” Univariate and multivariate analyses showed that the SCOPA-AUT score impacted the QoL (*p* = 0.001), especially the gastrointestinal (*p* = 0.007), and cardiovascular (*p* = 0.049) dimensions.

**Conclusion:**

Our data show that all patients have presented at least one NMS. Autonomic and sleep disorders were the most frequent, and in contrast to other studies, digestive and cardiovascular disorders were rather the factors influencing negatively the QoL of patients. Understanding the pathophysiology of these NMSs should be placed at the forefront in order to develop new therapeutic approaches by improving the QoL of PD patients.

## Introduction

Parkinson’s disease (PD) is one of the most prevalent neurodegenerative diseases and the number of patients is increasing. It is reported to be the second most common neurodegenerative pathology of the central nervous system (1). Its prevalence is estimated between 1 and 2 per 1,000 in unselected populations (2). The prevalence of the disease can be variable with age, as it’s affecting 1% of the general population above 60 years (3) and about 4% in highest age (4), and it is still a rare disease before the age of 50 (1, 5). PD is a complex neurological pathology characterized essentially by motor signs, including rest tremor, muscle rigidity, akinesia, and postural instability (6, 7). However, diverse non-motor symptoms (NMSs), such as sleep disorders, psychiatric disorders, autonomic disabilities, and sensory disturbances are also present in PD (8). These NMSs may contribute to the impairment of patient’s quality of life (QoL).

From a pathophysiological point of view, the PD motor symptoms are attributed to the degeneration of the dopaminergic nigrostriatal system (7). Nevertheless, increasing evidences have shown that PD is a multisystem disorder characterized also by the degeneration of the meso-cortical dopaminergic system, the noradrenergic system of the locus coeruleus, the serotonergic system of the dorsal raphe nuclei, and the cholinergic system of the nucleus basalis of Meynert, in addition to the histaminergic, peptidergic, and olfactory-related systems (8, 9).

During the past decades, understanding the pathophysiology of the motor symptoms was at the origin of the development of pharmacological and surgical therapies allowing their successful management (10–13). However, the knowledge of the NMSs pathophysiology is still limited. Recently, the importance given to the NMSs is increasing and it is now recognized that they may precede motor symptoms and may be useful in identifying potential patients to develop a neurodegenerative disease, such as PD (14, 15). Interestingly, clinical studies suggested that olfactive deficits, rapid eye movement sleep behavior disorder (RBD), fatigue, and depression may constitute important markers of preclinical stages of PD (16, 17).

From several reports, it appears that NMSs are estimated to occur at least in one-third of PD patients (18–20). However, their prevalence and relationship with the QoL in Moroccan patients are still unknown. This study aimed first to evaluate the prevalence of the NMSs in a cohort of 117 Moroccan parkinsonian patients and second to determine the impact of NMSs on their QoL.

## Materials and Methods

### Subjects

A cohort of 117 patients was gathered from different regions of Morocco in the department of Neurology and Neurogenetics in Ibn Sina University Hospital of Rabat (Hôpital des Spécialités de Rabat). All patients included in the study had a confirmed diagnosis of PD as per the UK Brain Bank Clinical Diagnostic criteria (21) and provided a written informed consent to take part in our study. The study was approved by the ethics committee of medical school of Rabat (CERB). Collected data included demographic characteristics, the medical history, the disease duration, presence of similar cases in the family, patients’ profession, and the socio-economic levels. Regarding the cognitive profile of our patients, individuals with a mini mental state examination (MMSE) score less than 21 were excluded to avoid interferences of cognitive impairment in NMS evaluation. The Moroccan version of the MMSE we use, considers the cut-off for dementia at 21 for illiterate patients or with low level of education. We divided the studied population into two groups: a group with low level of education [level 1–2 of international standard classification of education (ISCED)] and middle to higher level of education (22). We used some specific exclusion criteria, such as: co-morbidity which might limit walking ability (e.g., inflammatory arthropathy); serious hearing deficit, other neurological problems (stroke, inflammatory diseases), and acute medical problems (e.g., cardiopathy) that can also impact QoL.

### Clinical Evaluation of Motor Symptoms

The motor stage of PD was evaluated according to the UPDRS scale (I–VI) during ON condition. UPDRS I assess mentation, behavior, and mood (range 0–16); UPDRS II evaluates Activities of daily living (ADL), including speech, swallowing, handwriting, dressing, hygiene, falling, salivating, turning in bed, walking, and cutting food (range 0–52). UPDRS III is a score for motor examination (range 0–108). Each item of those scales scored on a scale from 0 to 4. UPDRS IV assesses the treatment’s complications in the week preceding the examination (items 32–35 for dyskinsia, items 36–39 for motor fluctuations, and items 40–42 for digestive, sleep, and autonomic complications). UPDRS V is the Hoehn and Yahr’s staging of severity of PD (staging 1–5), and UPDRS VI is Schwab and England’ to assess independency on activity of daily living on OFF and on ON conditions (we only assessed this scale during ON condition) (range 0–100% and higher scores refer to better functional independency).

The clinical type at onset of the disease was classified as tremor dominant, akinetic-rigid, or mixed form according to criteria used by Rajput et al. (23). Tremor dominant subtype referred to patients in whom the tremor was the dominant feature compared to bradykinesia and rigidity. Patients with prominent bradykinesia and rigidity with no visible tremor were classified as akinetic-rigid subtype and those who had comparable severity of bradykinesia, rigidity, and tremor were classified as mixed subtype. The first side and limb affected at the onset of the disease were also recorded. Levodopa equivalent daily dose (LEDD) was calculated based on Tomlinson et al. recommendations (24).

### Clinical Evaluation of Non-Motor Symptoms

The following symptoms were assessed: hyposmia, neuropsychiatric disorders (depression, anxiety, suicidal thoughts), autonomic dysfunctions (constipation and urinary dysfunctions), and sleep disturbances [excessive daytime sleepiness (EDS) and the quality of sleep]. A clinical interview was conducted to determine the presence of each NMS at the time of the examination. Informations on the current use of medications, such as laxatives, hypnotics, or antidepressants to treat some of these NMS, were also collected.

Smell loss was assessed by the validated Argentina Hyposmia Rating Scale (AHRS) (25) starting by asking the subjects whether they noted a change in their ability to smell. Patients with factors that could impair odor identification, such as: current smokers, medical history of nasal surgery (to correct a deviated septum or other plastic surgeries), allergic rhinitis, and traumatic nasal injuries were eliminated. According to these criteria, our sample for this test was restricted to 51 patients (23 males and 28 females). Hyposmia was considered to be present if the AHRS score was lower than 22.

To evaluate the presence of depression and its severity, we used the Montgomery–Asberg Depression Rating Scale (MADRS) (26). The cut-off scores we used were: <7 absent signs, mild signs = 7–18, moderate = 18–34, and ≥35 reflects severe depression. This scale consistently has the highest Cronbach’s alpha levels reaching 0.92 (27). Patients with severe depression (MADRS > 35) were excluded from the study. Patients with scores ≥7 were considered having depression.

The anxiety at the time of this study was evaluated using the Hamilton Anxiety Rating Scale (HAM-A). The cut-off scores we used were: mild anxiety = 8–14; moderate anxiety = 15–23; severe signs of anxiety ≥24; and scores ≤7 were considered to reflect no or minimal signs of anxiety (28). Patients with score ≥8 were considered having anxiety.

Dysautonomic symptoms were assessed using the self-reported autonomic symptoms in PD the SCOPA-AUT questionnaire (29). The 25 items of the SCOPA-AUT are grouped into 6 domains: gastrointestinal functioning (7 items), urinary functioning (6 items), cardiovascular functioning (3 items), thermoregulatory functioning (4 items), pupillomotor functioning (1 item), and sexual function (2 items for men and 2 for women). The score for each item ranges from 0 (where the patient had never experienced the symptom) to 3 (where the patient describes that the symptom is often experienced). The SCOPA AUT total score ranges from 0 to 75 (29).

The quality of sleep was appreciated using the Pittsburgh sleep quality index (PSQI) questionnaire (Dimension 1–7) (30). A total PSQI-score of 5 or greater was considered indicative of poor quality of sleep. EDS was evaluated using the Epworth sleepiness scale (31). A total Epworth score greater than 8 is indicative of excessive diurnal sleepiness and a score of 15 or greater represents a severe EDS.

The fatigue was assessed using the Fatigue severity scale (FSS), the subject is asked to read each statement and circle a number from 1 to 7, depending on how appropriate they felt the statement applied to them over the preceding week. A low value indicates that the statement is not very appropriate whereas a high value indicates agreement. The scoring is done by determining the average response to the questions (adding up all the answers and dividing by nine) (32).

The pain was evaluated by the Douleur neuropathique-4 (DN4) questionnaire, which consists on interviewing questions (DN4-interview) and physical tests; the test was considered positive for a score >4, and the Visual analog scale (VAS) was used to quantify the intensity of that pain. The scale is presented to the patient who is asked to place a line perpendicular to the VAS line at the point that represents his pain intensity. It ranges from 0 (no pain at all) to 10 (my pain is as bad as it could possibly be) (33).

In order to clarify whether the form of the disease could possibly influence the NMSs, we studied the correlation between both of them. For statistical constraints, we divided the patients into two groups: the tremoric dominant form group and the akinetic-rigid and mixed form as a second group.

### Quality of Life

We chose the 39-item PD questionnaire (PDQ39) to evaluate the QoL. It is a PD-specific health status questionnaire comprising 39 items. It contains eight domains: mobility, activities of daily living (ADL), emotional well-being, stigma, social support, cognition, communication, and bodily discomfort. The PDQ-39SI is obtained by the sum of the eight PDQ-39 scale scores divided by eight, which gives a score between 0 and 100 (“0% = no difficulties” to “100% = maximum level of difficulty”). Higher scores indicated poorer QoL (34).

We analyzed the correlation between NMSs and the QoL of Moroccan patients with PD. After specifying the most affecting NMS on the QoL scores, we analyzed its impact on each of the eight dimensions of the PDQ39 questionnaire.

We also compared the differences between men and women regarding their QoL scores and identified the most affected dimension for each gender.

### Statistical Analysis

Demographic and clinical variables were analyzed using parametric and nonparametric tests as appropriate using SPSS 13.0 software. Quantitative data were expressed as mean ± SD or median and interquartile range. Categorical variables were expressed as numbers and percentages.

The comparison of NMS’s prevalence between clinical phenotypes (tremoric dominant form versus akinetic-rigid and mixed form) was done using logistic regression.

To eliminate the possible confounding factor we assessed, in the univariate analysis, the relationships between clinical form and age, gender, disease duration, LEDD, UPDRS I, UPDRS II, UPDRS III, UPDRS IV, UPDRS V, UPDRS VI, PDQI, FSS, Epworth, DN4, VAS, PDQ39, SCOPA-AUT, MADRS, HAM-A, MMSE, AHRS. In the multiple regression, we adjusted for the variables that were statistically significant in the multivariate analysis (*p* < 0.05) (LEDD, UPDRS IV, UPDRS I, UPDRS II, UPDRS III, UPDRS IV, UPDRS VI, FSS, VAS, PDQ39, SCOPA-AUT, MADRS, HAM-A).

In order to identify factors that can impact the QoL we assessed the relationship between PDQ39 and the other variables (age, level of education, gender, disease duration, LEDD, UPDRS I, UPDRS II, UPDRS III, UPDRS IV, UPDRS V, UPDRS VI, SCOPA-AUT, Epworth, PSQI, MADRS, HAM-A, MMS, DN4, VAS, FSS). The correlation was determined, in the univariate analysis, using the Spearman test for quantitative variables and Mann–Whitney test for gender and level of education. Linear regression was used in the multivariate analysis. We adjusted for disease duration (*p* = 0.065) and variables that were statistically significant in the univariate linear regression (*p* < 0.05) (disease duration, UPDRS I, UPDRS VI, PSQI, FSS, VAS, SCOPA-AUT, HAM-A, MADRS). We did not introduce the VAS score (*p* < 0.001 in the univariate analysis) in the multivariate model because it has strong collinearity with the DN4.

The relationship between the dimensions of PDQ39 score and SCOPA-cardiovascular, SCOPA-gastrointestinal was analyzed using Spearman test.

Categorical variables were expressed as numbers and percentages and were compared using Chi-square test. For multiple testing, we corrected the p value by the Bonferroni method.

## Results

### Demographic Data and Motor Aspects

We enrolled into this study a total of 117 patients who fulfilled the inclusion and exclusion criteria of PD. The demographic features are reported in Table 1.

**Table 1:** Characteristics of Moroccan Parkinson’s disease (PD) patients.

Variables	PD patients (*n* = 117)
Age (years)	60.77 ± 11.36^b^
Age at onset (years)	54.28 ± 12.019^b^
Level of education	
• Low level	69.2% (81)
• Middle to high	30.8% (36)
Disease duration (years)	6 [2.5–9.5]^a^ 6.65 ± 5^c^
Gender (male) in%	55.6% (65)
Clinical form in%	
• Trembling	39.3% (46)
• Akinetic-rigid	20.5% (24)
• Mixed	40.2% (47)
Side of the disease’s onset in%	
• Right	54.7% (64)
• Left	42.7% (50)
• Bilateral	2.6% (3)
LEDD (mg/day)	325 [200–500]^a^
	379.41 ± 266.61^c^
UPDRS I	3 [1–4]^a^
	2.73 ± 2.1^c^
UPDRS II	8 [4–13]^a^
	9.61 ± 7.84^c^
UPDRS III	14 [7–29]^a^
	18.98 ± 16.70^c^
UPDRS IV	3 [0.25–6]^a^
	3.73 ± 3.84^c^
Hoen and Yahr (>2) in%	27.3% (32)
Schwab and England in%	90% [80–90]^a^
MMSE	25.92 ± 3.80^b^

*^a^Median and interquartile ranges*.

*^b^Mean ± SD*.

*^c^Indicative means for parameters with non-normal distribution*.

*LEDD, levodopa equivalent daily dose; UPDRS, unified Parkinson’s disease rating scale; MMSE, mini-mental state examination*.

The overall mean age of this study population was 60.77 ± 11.36 years. The mean age at onset of the disease was 54.28 ± 12 years and the median of the disease duration was 6 [2.5–9.5] years. The study cohort included 65 (55.6%) males and 52 (44.4%) females. 81 of our patients (69.2%) had low level of education.

Our patients presented different clinical forms with 40.2% (*n* = 47) of the mixed akinetic-rigid-tremoric form, 39.3% (*n* = 46) of the tremor-dominant form, and 20.5% (*n* = 24) of the akinetic-rigid form. The onset of the motor symptoms affected mainly the right side [54.7% (*n* = 64)] and in 2.5% (*n* = 3) of cases the onset was bilateral with asymmetry. The median LEDD was 325 mg per day [200–500].

The median UPDRS-III score was 13 [7–27.75]. The median score of Hoehn and Yahr was 2 [1–2.5] and 27.3% (*n* = 32) of cases had a score above 2. The median score of Schwab and England during ON condition was 90% [80–90%] (Table 1).

### Non-Motors Aspects

All our patients (100%) presented at least one NMS. The prevalence of NMS and the medians of the scores used to assess them are presented in Table 2. Regarding the clinical phenotype, it did not impact the prevalence of any of the NMSs (Table 3). The univariate analysis showed significant results for some NMSs (depression, anxiety, fatigue, and dysautonomic symptoms) and the PDQ39. However, none of these parameters was significant in the multivariate analysis.

**Table 2:** Prevalence of non-motor features of Parkinson’s disease (PD) patients.

Variables	Prevalence%	Missing data	Global scores
**Autonomic symptoms**			
Gastrointestinal dysfunctions (SCOPA:1–7)	80 (92)	2	3 [1–7]^a^ 4.54 ± 4.13^c^
Urinary dysfunctions (SCOPA: 8–13)	82.6 (95)	2	5 [2–8]^a^ 5.20 ± 4.32^c^
Cardiovascular dysfunctions (SCOPA:14–16)	50.9 (59)	1	1 [0–2]^a^ 1.39 ± 1.84^c^
Thermoregulatory dysfunctions (SCOPA:17–20)	58.6 (68)	1	1 [0–3]^a^ 2.28 ± 3.09^c^
Pupillomotor dysfunctions (SCOPA: 21)	31 (36)	1	0 [0–1]^a^ 0.58 ± 0.99^c^
Sexual dysfunctions (SCOPA: 22–23)	47.9 (34)	46	0 [0–4]^a^ 1.7 ± 2.18^c^
Scopa AUT: total score			14 [7.75–21.08] 15.5 ± 11.08^c^

**Sleep symptoms**			
Poor sleep quality (PSQI > 5)	80.6 (87)	9	8 [6–12.75]^a^ 9.48 ± 4.72^c^
Excessive daytime sleepiness (Epworth > 10)	23.4 (25)	10	5 [2.28–9.80]^a^ 6.38 ± 5.35^c^

**Psycho-cognitive syndromes**			
Anxiety (HAM-A > 7)	50.9 (58)	3	8 [3–13.25]^a^ 9.46 ± 8.09^c^
Depression (MADRS > 6)	47.9 (56)	0	6 [2–12] 7.77 ± 6.58^c^
MMSE			25.89 ± 3.84^b^

**Miscellaneous**			
Fatigue (FSS > 5.25)	23.1 (27)	0	3.22 [1.5–5.05]^a^ 3.52 ± 2.01^c^
Pain (DN4 > 3)	11.1 (13)	0	1 [0–2]^a^ 1.22 ± 1.85^c^
VAS	28 (14)	67	0 [0–5]^a^ 2.17 ± 2.69^c^
Olfactory changes (AHRS < 22)			24 [20–24]^a^ 20.32 ± 6.39^c^

*% (number of patients)*.

*^a^Median and interquartile ranges*.

*^b^Mean ± SD*.

*^c^Means for parameters with non-normal distribution*.

*HAM-A, Hamilton anxiety scale for anxiety; MADRS, Montgomery–Asberg Depression Rating Scale for depression; HAM-A, Hamilton anxiety rating scale; SCOPA-AUT, self-reported autonomic assessment for Parkinson’s disease; VAS, visual analog scale; DN4, “Douleur neuropathique 4” questionnaire for pain; Epworth, scale for excessive daytime sleepiness; FSS, fatigue severity scale; PSQI, Pittsburg sleep quality index; MMSE, mini-mental scale examination; PDQ39-SI, Parkinson’s disease questionnaire 39 score index; AHRS, Argentina hyposmia rating score*.

**Table 3:** Comparison of non-motor signs between trembling form group and akinetic-rigid form and mixed form group.

Variable	Trembling form	Akinetic-rigid and mixed form	Univariate analysis *p*-value	Multivariate analysis		
				β	CI 95%	*p*
Age	61.73 ± 11.38	60.15 ± 11.39	0.460			
Gender (male)	58.7 (27)	53.5 (38)	0.582			
Disease duration	4 [2–8]	6 [4–10]	0.092			
LEDD	225 [150–412.5]	400 [224.36–550]	0.014	1.002	1–1.004	0.078
UPDRS IV	1 [0.00–3.25]	4 [2–7]	0.002	1.013	0.844–1.217	0.887
UPDRS I on	2 [1–2]	3 [1–4]	0.021	1.073	0.922–1.249	0.447
UPDRS VI on	90% [80–100%]	80% [70–90%]	0.001	1.208	0.875–1.666	0.367
UPDRS II	4 [3–9]	11 [5–17]	0.001	1.019	0.971–1.069	0.360
UPDRS III	11 [6–17.5]	17 [7–32]	0.003	1.267	0.758–2.119	0.447
UPDRS V	Stage 2 [stages 1–2]	Stage 2 [stages 1–2]	0.083			
PSQI	7 [6–11.5]	9 [6–13]	0.122			
FSS	2.77 [1–4]	4.11 [2–6]	0.001	1.208	0.875–1.666	0.251
Epworth	5 [1–9]	5.1 [2.9–11.4]	0.174			
DN4	0 [0–1]	1 [0–2]	0.059			
VAS	0 [0–.25]	2 [0–5]	0.003	1.099	0.891–1.356	0.379
PDQ39	17.23 [6.77–6.39]	30.36 [17.1–39.47]	0.001	0.999	0.999–0.951	0.959
SCOPA-AUT	11.5 [6–7]	15.16 [9.2–21.97]	0.041	0.978	0.914–1.047	0.527
MADRS	4 [0–8]	8 [5–14]	0.003	1.069	0.937–1.220	0.318
HAM-A	6 [1–0.5]	10 [4–14]	0.020	0.986	0.868–1.121	0.830
MMSE	26.5 ± 3.79	25.49 ± 3.85	0.176			
AHRS	24 [17–24]	24 [20–24]	0.663			

*CI, confidence interval; LEDD, levodopa equivalent daily dose, UPDRS, Unified Parkinson’s disease rating scale; PSQI, Pittsburg sleep quality index; FSS, fatigue severity scale; Epworth, scale for excessive daytime sleepiness; DN4, “Douleur neuropathique 4” questionnaire for pain; VAS, visual analog scale; SCOPA-AUT, self-reported autonomic assessment for Parkinson’s disease; MADRS, Montgomery–Asberg Depression Rating Scale for depression; HAM-A, Hamilton anxiety scale for anxiety; MMSE, mini-mental scale examination; PDQ39-SI, Parkinson’s disease questionnaire 39 score index; AHRS, Argentina hyposmia rating scale*.

#### Autonomic Dysfunctions

The median score of SCOPA-AUT test was 14 [7.75–21.80]. A large number of our patients suffered from urinary [*n* = 95 (82.6%)] and gastrointestinal dysfunctions [*n* = 92 (80%)]. Sexual impairments were observed in 49.3% (*n* = 34), cardiovascular dysfunctions in 50.9% (*n* = 59), thermoregulatory troubles in 58.6% (*n* = 68), and pupillomotor dysfunctions in 31% (*n* = 36) of cases. Patients presented at least two autonomic disorders at the same time in 86.3% (*n* = 101) of cases and only 2.5% (*n* = 3) had no autonomic dysfunctions (Table 2).

#### Sleep Disorders

Sleep disorders were the second more frequent symptoms in our cohort after urinary disturbances with 80.6% (*n* = 87) of cases experiencing poor quality of sleep. The median PSQI score was 8 [6–12.75]. All those patients used hypnotics. The median score of Epworth score for excessive daily sleepiness was 5 [2.28–9.8] with 23.4% (*n* = 25) of our patients having EDS, among which 6.4% had severe EDS (Epworth score ≥16).

#### Neuropsychiatric Symptoms

Depressive symptoms were present in 47.9% (*n* = 56) of patients, 12.5% (*n* = 7) had moderate depression, and 87.5% (*n* = 49) mild depression. The median MADRS score was 6 [2–12]. Anxiety disorders were present in 50.9% (*n* = 58) of our patients with a median score of 8 [3–13.25]. In these patients, 55.1% (*n* = 32) had mild signs, 32.7% (*n* = 10) had moderate, and 12% (*n* = 7) had severe sings of anxiety. About 11% of our patients were treated for depression (*n* = 6) and 12% (*n* = 7) were treated for anxiety. About 7% (*n* = 8) of all the 117 patients had suicidal thoughts, 6% (*n* = 7) reported transient suicidal thoughts, and less than 2% (*n* = 2) only believed that suicide was considered as a possible solution.

#### Fatigue and Pain

About the quarter of our patients (23.1%, *n* = 27) suffered from fatigue and the median FSS was 3.22 [1.5–5.05] and only 11.1% (*n* = 13) suffered from neuropathic pain with a VAS >5.

#### Olfaction

Only 50/117 (42.7%) patients were submitted to the AHRS test and their median score was 24 [20–24]. In these patients, 86% (*n* = 43) were aware of their smell loss, but only 28% (*n* = 14) of them had a score below 22 and 14% (*n* = 7) had severe scores (<10). The patients with a positive score estimated that the olfactory changes appeared before the manifestation of motor symptoms. The estimated duration was different from a patient to another with a median less than 1 year and ranges from 10 months to 20 years. Their mean age was 67.7 ± 8 years old vs 58.63 ± 1 for those without olfactory changes (*p* = 0.009). The side of onset of PD in those patients was equal between right and left sides (46.7% each) while 6.7% started the disease bilaterally with asymmetry.

#### QoL and Its Correlation With NMSs

The median score of the PDQ39-SI was 23.22 [13.36–36.69]. The scores of the PDQ39-SI dimensions are presented in Table 4. Mobility was the highest score with a median of 30 [11.25–57.5]. Regarding the non-motor dimensions, emotional well-being had a median score of 25 [8.3–54.16], cognition 25 [12.5–41.18], bodily discomfort 25 [0–50] ranking the top. Social support was the last score with a median of 0 [0–16.6].

**Table 4:** Dimensions of the PDQ39 in the study population.

PDQ39 SI and its dimensions	All patients	Males	Females	*p*
PDQ39-SI	23.22 [13.36–36.69] 25.83 ± 16.54	19.42 [11.09–35.72] 23.42 ± 16.13	26.61 [16.34–39.27] 28.83 ± 16.71	0.070

Mobility	30 [11.25–57.5] 35.29 ± 27.86	25 [10–40] 27.5 ± 23.39	53.75 [13.12–71.87] 45.04 ± 30.07	**0.002**

ADL	20.88 [0–39.58] 25.35 ± 24.71	20.88 [4.16–43.75] 26.41 ± 25.46	16.66 [0–36.45] 24.03 ± 23.92	0.633

Emotional well-being	25 [8.3–54.16] 32.62 ± 27.50	25 [4.16–35.41] 25.96 ± 23.87	37.5 [12.5–65.62] 40.94 ± 29.63	**0.005**

Stigma	18.75 [0–50] 26.88 ± 28.45	21.87 [0–50] 29 ± 29.40	12.5 [0–42.18] 24.29 ± 27.30	0.370

Social support	0 [0–16.6] 10.27 ± 18.137	0 [0–8.33] 7.94 ± 15.67	0 [0–16.66] 13.14 ± 20.56	0.121

Cognition	25 [12.5–41.18] 26.83 ± 22.31	21.87 [0–37.5] 23.73 ± 21.99	25 [12.5–50] 30.64 ± 22.32	0.081

Communication	8.3 [0–31.25] 17.81 ± 22	8.33 [0–33.33] 17.96 ± 22	8.33 [0–25] 17.62 ± 22.18	0.922

Bodily discomfort	25 [0–50] 32.54 ± 29.14	25 [0–50] 30.59 ± 28.21	25 [8.33–66.66] 34.93 ± 30.34	0.520

*Values are expressed as median and interquartile ranges as distribution was non-Gaussian and corresponding means and SD are presented below each median*.

*PDQ39, Parkinson’s disease questionnaire 39; ADL, activity of daily living*.

*Bold font means significant p values*.

We compared the PDQ39-SI scores between men and women. The median score of PDQ39-SI for women was 26.61 [16.34–39.27] vs 19.42 [11.09–35.72] for men, with no significant difference (*p* = 0.070). The only significant differences between the two groups when comparing the eight dimensions of this score were in the well-being and mobility, where women had a worst score (*p* = 0.005 and *p* = 0.002, respectively) (Table 4).

We also investigated the relationship between level of education and PDQ 39 SI and there was no difference in the PDQ39 SI score between the two groups (*p* = 0.398).

The univariate analysis of the correlation between the PDQ39 and other assessments are listed in the Table 5. Of interest, is that age, MMSE score, the daytime sleepiness, and the AHRS showed no correlations with the QoL while motor signs were well correlated as predicted. Moderate positive correlation was found between the PDQ39 and LEDD and SCOPA-AUT sexual dysfunction score (0.01 < *p* < 0.05). Strong positive correlation (*p* < 0.001) was found with the disease duration and the following NMSs-scores: UPDRSI, HAM-A, MADRS, DN4, PSQI, FSS, SCOPA-AUT gastrointestinal, urinary, cardiovascular, thermoregulatory, and pupillomotor dysfunctions.

**Table 5:** Correlation between motor and non-motor features and the PDQ39-SI (Spearman test).

Variable	Correlation coefficient
Age	0.044
Disease duration	0.287**
LEDD	0.228*
UPDRS IV	0.464**
UPDRS I on	0.491**
UPDRS II on	0.654**
UPDRS III on	0.253**
UPDRS VI on	0.487**
SCOPA-AUT gastrointestinal dysfunctions	0.448**
SCOPA-AUT urinary dysfunctions	0.322**
SCOPA-AUT cardiovascular dysfunctions	0.368**
SCOPA-AUT thermoregulatory dysfunctions	0.455**
SCOPA-AUT pupillomotor dysfunctions	0.239**
SCOPA-AUT sexual dysfunctions	0.253*
SCOPA-AUT	0.597**
PSQI	0.476**
Epworth	0.103
MADRS	0.619**
HAM-A	0.570**
MMSE	−0.170
FSS	0.548**
DN4	0.262**
VAS	0.359**
AHRS	−0.231

*p < 0.05.

**p < 0.01.

*PDQ39-SI, Parkinson’s disease questionnaire 39 score index; LEDD, levodopa equivalent daily dose; UPDRS: unified Parkinson’s disease rating scale; PSQI, Pittsburg sleep quality index; FSS, fatigue severity scale; Epworth, scale for excessive daytime sleepiness; DN4, “Douleur neuropathique 4” questionnaire for pain; VAS, visual analog scale; SCOPA-AUT, self-reported autonomic assessment for Parkinson’s disease; MADRS, Montgomery–Asberg Depression Rating Scale for depression; HAM-A, Hamilton anxiety scale for anxiety; MMSE, mini-mental scale examination; AHRS, Argentina hyposmia rating scale*.

In the multivariate analysis, the correlation between NMSs and PDQ39-SI (Table 6) aimed to see which NMS did affect the QoL of our patients. It showed that the most correlated ones were the autonomic dysfunctions (*p* < 0.001), especially the gastrointestinal *(B* = 1.067 [0.391–1.743], *p* = 0.007) and cardiovascular symptoms (*B* = 1.067 [0.391–1.743], *p* = 0.49). Constipation was the most frequent gastrointestinal sign (62.9%, *n* = 73) and swallowing difficulties was the least frequent complaint (23.3%, *n* = 27). Orthostatic hypotension is the cardiovascular sign sought by the SCOPA-AUT questionnaire, signs of light-headed after passing from sitting to standing position were present in 42.2% of cases (*n* = 49) and only 15.5% (*n* = 18) had fainted during the 6 months prior to the questionnaire. Table 7 shows the results of the correlation between the PDQ39 dimensions and the most disabling autonomic dysfunctions (gastrointestinal and cardiovascular symptoms). Cardiovascular symptoms did not correlate with all the questionnaire’s dimensions. Gastrointestinal had positive correlation with all dimensions except emotional well-being.

**Table 6:** Correlation between non-motor signs and PDQ39-SI (linear regression and multiple linear regression).

Variable	Univariate analysis *p*-value	Multivariate analysis		
		β	CI 95%	*p*
Age	0.642			
Gender	0.079			
Disease duration	0.023	−0.155	−0.673–0.363	0.552
LEDD	0.074			
UPDRS II + III + IV	0.001	0.096	−0.049–0.240	0.191
UPDRS VI ON	0.001	1.326	−0.611–3.263	0.177
SCOPA-AUT	0.001	0.591	0.323–0.859	<0.001
PSQI	0.001	0.367	−0.243–0.976	0.235
Epworth	0.200			
MADRS	0.001	0.372	−0.182–0.926	0.186
HAM-A	0.001	0.160	−0.326–0.645	0.515
UPDRS I	0.001	0.654	−0.741–2.050	0.354
MMSE	0.001	0.002	−0.671–0.675	0.995
FSS	0.001	0.297	−1.184–1.779	0.691
DN4	0.001			
VAS	0.001	0.037	−1.005–1.078	0.944

*CI, confidence interval; LEDD, levodopa equivalent daily dose; UPDRS, unified Parkinson’s disease rating scale; PSQI, Pittsburg sleep quality index; FSS, fatigue severity scale; Epworth, scale for excessive daytime sleepiness; DN4, “Douleur neuropathique 4” questionnaire for pain; VAS, visual analog scale; SCOPA-AUT, self-reported autonomic assessment for Parkinson’s disease; MADRS, Montgomery–Asberg Depression Rating Scale for depression; HAM-A, Hamilton anxiety scale for anxiety; MMSE, mini-mental scale examination; PDQ39-SI, Parkinson’s disease questionnaire 39 score index*.

**Table 7:** Correlation between the PDQ39 dimensions and the most disabling autonomic items gastrointestinal, cardiovascular symptoms.

PDQ39 Dimensions	SCOPA-AUT cardiovascular score	SCOPA-AUT Gastrointestinal score
	
	Correlation coefficient/*p*	
Mobility	0.307**	0.367**
ADL	0.315**	0.363**
Emotional well-being	0.444**	0.174
Stigma	0.177	0.248**
Social support	0.395**	0.212*
Cognition	0.182	0.336**
Communication	0.176	0.403*
Bodily discomfort	0.253**	0.314**

*p < 0.05.

**p < 0.01.

*PDQ39, Parkinson’s disease questionnaire 39; SCOPA-AUT, self-reported autonomic assessment for Parkinson’s disease, ADL, activity of daily living*.

## Discussion

This is the first study on NMSs in PD patients enrolled in Morocco. Our results show a high prevalence of NMSs in our patients, in whom at least one NMS has been found. Our results are in line with previous studies (35, 36), in which the authors have found a NMS prevalence rate of 100% in a cohort of 82 and 134 patients, respectively.

In our study, the incidence of PD was slightly higher in male than in female and the average age of our patients was 60.77 years, which is in line with the international reports. By focusing on the clinical manifestations, we found that the most common type of onset was the mixed type and the tremor predominant type with a very slight difference, while the akinetic-rigid form was less common. However, the clinical form did not impact the NMSs. Screening of NMSs showed that the autonomic symptoms are the most frequent, followed by sleep and psychiatric (depression) disorders. Fatigue and neuropathic pain have been the less common NMSs present in our patients.

The most frequent NMSs were the urinary disturbances, which is in line with several previous studies (20, 37–39). Urinary disturbances reported in PD patients are diverse and their prevalence through studies is different. The most reported symptoms are: urge incontinence (39, 40), neurogenic detrusor over-activity (40, 41), detrusor hyperreflexia (42), and nocturia (43).

The gastrointestinal symptoms were present in 80% of our patients. Using the SCOPA-AUT scale we were investigating different gastrointestinal symptoms, such as swallowing difficulties, excessive salivation, constipation, and difficult defecation. Constipation was the most common symptom in our patients and swallowing difficulties were around 20% which is in line with previous reports (44, 45). More other symptoms like gastroparesis, gastroesophageal reflux, or weight loss are also frequent in all the stages of the disease course, as suggested by the Braak’s theory for the pathodynamics of Lewy pathology in PD, and considered as premotor symptoms (46–48). All these symptoms affect at least one-third of the patients (49). Being one of the first organs where the alpha-synuclein is deposited, these symptoms reflect the dysfunction of the enteric nervous system and the stomach, or are considered as side effects of the antiparkinsonian drugs (49). In their study, Cersosimo et al. (49) showed that the gastrointestinal symptoms reported to occur before the onset of motor symptoms were constipation in 87% of patients, defecatory dysfunction in 58.9%, and dry mouth in 20.5%. The fact that their prevalence was similar to that of controls makes this outcome debatable.

While gastrointestinal and urinary dysfunctions usually occur early in PD, cardiovascular symptoms tend to appear and to become prominent with the progression of the disease (50). Some authors associate cardiovascular symptoms to sympathetic dysfunctions and suggest that they may appear in earlier stages even in drug-naïve patients or precede the appearance of motor symptoms (50–52). Cardiovascular symptoms (orthostatic hypotension) were present in more than half of our patients, which is in line with some previous reports (39).

Sexual dysfunctions were present 49.3% of our patients, but we believe that this percentage does not represent the reality of genital troubles that our patients face and it is rather a cultural finding, and consequently it is improper to compare it to other studies. Assessing these symptoms was mostly difficult. Our patients tended to be reserved regarding their sexual life, and those who answered tended to trivialize the existing troubles and relate them to their advanced age. In a previous study, Chaudhuri et al. (45) reported that the questions relating to sex were frequently left unanswered.

Sleep dysfunctions are prevalent in PD and represent an integral component of the disease, which may emerge over different phases of PD. It could be the result of the disease itself, secondary to other NMSs, or a side effect of medicines. It is affecting 42–98% of PD patients (53, 54). RBD is the most typical sleep disorder associated with PD, but fragmented sleep in PD, which is multifactorial is commonly reported (55). In addition, patients with PD are commonly affected by primary sleep disorders also existing in the general population in particularly restless-legs syndrome (RLS, also known as Willis–Ekbom disease) or periodic leg movements in sleep and sleep disordered breathing, notably obstructive sleep apnea (56). The regulation of the normal sleep-wake cycle has been shown to involve the pedunculopontine nucleus (PPN) whose role is paramount. In PD, the PPN cholinergic neurons showed special selective vulnerability (57). In our cohort, a large number of patients (80.6%) suffered from poor sleep quality while only 23.4% had EDS. Previous studies showed that 50% of PD patients had prolonged daytime sleepiness or unconscious drowsy (58). This prevalence is much higher than in our study, which can be due to the inadequacy of the Epworth scale in our population as two items are not applicable for illiterate patients. Another explanation can be the early stage of the disease of our patients (72.6% had a Hoehn and Yahr stage <2) and the exclusion of patients with dementia from our study. The frequency of EDS in PD seems to correlate with disease severity, treatment duration, and additional symptoms like dementia and depression (59–61).

The incidence of mental and behavioral disorders in PD patients is generally low except for depression (41.5%), apathy (40.9%), and anxiety (39.4%) (58). Depression may occur at any time during the course of PD, or precede the manifestation of motor symptoms by 4–6 years (62). It may appear early in the Braak stage II, when Lewy bodies deposit in dorsal raphe nucleus and locus coeruleus, and thus may precede the motor symptoms. It is generally attributed to noradrenaline and serotonin deficiency (63, 64). However, depressive symptoms are definitely increasing with the disease progression. Compared to the general population, depression in PD patients is two- to threefold more prevalent (62). Our data confirm those of previous studies showing a prevalence of depression up to 47.9%. The dominant character of depressive signs in our cohort was rather mild depressive symptoms. Suicidal thoughts were present only in 8% of patients. This can be explained by the impact of religion in our society, helping patients to better accept their chronic pathologies.

Concerning anxiety, our results are in line with previous reports. Its estimated prevalence in PD is 25–40% (65) and it is considered as a wearing off phenomenon. As for depression, anxiety can appear at any stage in the course of the disease and it may also precede the motor signs. In regards to the semiological presentation of anxiety in PD, it is usually seen as a generalized anxiety disorder, phobic disorder, or panic disorder (65).

Regarding olfactory disorders, they exist in a large majority of PD patients (up to 90%) and most often are present at the time of diagnosis. Though, in more than 70% of cases, patients are unaware of their smell changes (66). Smell impairment is considered a marked warning of PD before motor symptoms, which is due to the early involvement of olfactory related brain regions by alpha-synuclein according to Braak staging and has a good clinical predictive value for PD (46, 57). While some studies concluded that olfactory disturbances are independent of disease duration and stage (66, 67), others thought that they are associated with greater disease severity and progression and that severe hyposmia in individuals with PD can predict the course toward PD dementia (68). In our cohort, olfactory disorders were associated with a later age of diagnosis of the disease (67.5 years old), which is considered to be a predictor for functional dependency in PD (69). Some of our patients reported that the olfactory changes appeared years before the onset of motor symptoms. We used the AHRS (25), being simple and time conserver. It revealed 28% of patients with smell disorders, while 86% assumed that they were suffering from smell changes, which is making us questioning the sensitivity of this scale.

The fatigue, also described as a feeling of tiredness or exhaustion, has been increasingly known in the context of PD. Fatigue is mostly reported by PD patients as one of their most invalidating symptoms with the utmost negative effect on their QoL (70). 23.1% of our patients suffered from fatigue with a median score of 3.22. The pathophysiology of fatigue in PD is unknown, and no effective treatment exists. Goldman et al. reported that fatigue is associated with worse cognitive impairment (71) which can explain the poor frequency of fatigue recorded in our study as patients with dementia were excluded.

Pain was at the bottom of the NMSs frequency. Only 11.1% of our patients reported neuropathic pain. The prevalence of different pain types in PD varies from one study to other; it is commonly reported in the PD-NMSs studies in many various types: musculoskeletal, dystonic, or radicular central neuropathic pain. Their origin seems to be multifactorial, but responding well to Levodopa treatment (72–74). Santos-Garcia et al. (75) found that depression was an independent predictor of pain in PD patients, this may explain our results, since our patients presented mainly mild depression.

Despite the important prevalence of NMSs, the QoL of our patients was not strongly affected (median score at 23.22). Mobility dimension ranked the top of scores with a median of 30, while the median score of ADL was 20.88, which is expected, since PD constitutes primarily a motor handicap. The most affected non-motor dimensions of the PDQ39-SI in our patients were emotional well-being, bodily discomfort, and cognitive impairment. This is in line with the numerous studies that showed the adverse impact of depression and dementia on the QoL of PD patients (20, 76).

The least affected dimension was the social support. Indeed, the majority of our patients were well surrounded by their family members who provided support for their appointments. Stigma median score reached only 18.75%, we believe that our patients seemed to accept their disease and in the majority of cases do not feel embarrassed to reveal it.

In the correlation analysis, all the NMSs were correlated to the QoL, except for the EDS and olfactory changes. Although urinary symptoms were the most common in our patients, the multivariate analysis showed no correlation between urinary symptoms and the QoL. Surprisingly, gastrointestinal and cardiovascular symptoms had the most negative impact on the QoL of our patients. In the literature, the symptoms influencing the QoL of PD patients are diversely reported. A large number of studies reported fatigue, sleep disturbances, depression, apathy, and mood to be determinants of the QoL (38, 62, 77). Generally, depression is considered as the most important neuropsychiatric symptom impacting the QoL in PD patients (20, 37, 78–80), which is not found in our cohort. In line with our results, Svetlana Tomic et al. (39) found gastrointestinal symptoms to be impacting negatively the QoL of PD patients in a study focusing only on autonomic dysfunctions. Also, Gallagher et al. (37) reported QoL to be influenced by thermoregulatory, gastrointestinal, cardiovascular dysfunctions (in particular orthostatic hypotension), and urinary symptoms.

In this study, we analyzed the impact of those symptoms on each dimension of the PDQ39-SI and found that the gastrointestinal symptoms affected all the aspects of QoL (except for the emotional well-being). For cardiovascular symptoms, they did not influence cognition and communication, nor the stigma dimension, they affected negatively all the other aspects. These findings address the fact that before increasing dopaminergic drugs for patients’ complaints of difficulty in ADL or reduced mobility, one has to exclude the presence of NMSs that do impact mobility and treating them will in some extent, improve motor state of the patients.

Regarding the gender differences, our female patients had a tendency of higher PDQ39-SI than men but the difference was not significant. There was no significant difference between male and female in our study regarding the assessment of their QoL, except for the emotional well-being and mobility dimensions. Tomic et al. (39) reported the same findings especially that their study was only regarding the autonomic dysfunctions, in addition to this Hristova et al. (81) reported also women to present worst scores in social support and bodily discomfort. In the majority of the few studies regarding the QoL in PD, women seem to experience worst QoL than men (82). On top of that, Moore et al. (83) concluded in their study done on 100 PD patients that gender identity might have a significant effect on their QoL. The androgynous group (men and women) had the best QoL and furthermore, androgynous women had the best scores of all groups (83).

## Study Limitations

The study has some methodological limitations that we took into consideration when interpreting the findings. In other hand, our patients had low disease severity (27.3% had a Hoen and Yahr score >2) and the cognitive impact on QoL was not assessed as we excluded patients with dementia. Also, our study is cross-sectional, so the analysis was based on clinical data collected at a single point in time; therefore, any pattern of progression of the disease could not be estimated.

## Conclusion

The prevalence of NMSs in our study was 100%, as at least one NMS has been found in all our patients. Urinary symptoms were the most prevalent, although, gastrointestinal and cardiovascular symptoms were the factors that impacted the QoL. Our patients may underestimate the gastrointestinal symptoms without relating them to PD, especially if they exist long time before the onset of the motor symptoms. Also, cardiovascular symptoms constitute a real burden for our patients. These symptoms have to be investigated systematically and treated once present to improve the QoL of PD patients. More studies are needed to understand the pathophysiology of NMSs in order to develop new and effective therapeutic approaches.

## Ethics Statement

This study was carried out in accordance with the recommendations of the ethics committee of medical school of Rabat (CERB) with written informed consent from all subjects. The protocol was approved by the ethics committee of medical school of Rabat (CERB).

## Author Contributions

HT designed the study, collected and analyzed the data, wrote and edited the manuscript. KB assisted with collection and analysis of the data. AB, EH, AB, and MY agreement for clinical data. WR and AB contributed to the conception and supervision of the work and participated in writing the paper, edited and approved the final version to be published. All authors read and approved the final manuscript.

## Conflict of Interest Statement

The authors declare that the research was conducted in the absence of any commercial or financial relationships that could be construed as a potential conflict of interest.
